# Automated sirulated distillation using an articulated laboratory robot system

**DOI:** 10.1155/S1463924694000258

**Published:** 1994

**Authors:** William F. Berry, Vince Giarrocco

**Affiliations:** Hewlett-Packard Company 2850 Centerville Road Wilmington DE 19808-1610 USA

## Abstract

An automated method, based on the Hewlett-Packard ORCA (Optimized Robot for Chemical Analysis) system, for sample preparation and analysis of petroleum samples by simulated distillation (SIMDIS) is described. Results obtained for the robotically prepared samples show excellent agreement with those obtained from the same samples prepared manually. The application, based on ASTM method D 2887, is the foundation for a more fully automated system that can perform a variety of SIMDIS samples and methods.


Journal of Automatic Chemistry, Vol. 16, No. 6 (November–December 1994), pp. 205–209

### Automated simulated distillation using an articulated laboratory robot system

**William F. Berry and Vince Giarrocco**

*Hewlett-Packard Company, 2850 Centerville Road, Wilmington, DE 19808-1610, USA*

*An automated method, based on the Hewlett-Packard ORCA (Optimized Robot for Chemical Analysis) system, for sample preparation and analysis of petroleum samples by simulated distillation (SIMDIS) is described. Results obtained for the robotically prepared samples show excellent agreement with those obtained from the same samples prepared manually. The application, based on ASTM method D 2887, is the foundation for a more fully automated system that can perform a variety of SIMDIS samples and methods.*

### Introduction

Sample preparation has been shown to be a bottleneck in most analytical processes. It contributes significantly to the total analysis time and to the errors that may occur within a procedure [1]. Automation can address these issues by increasing the consistency and reliability of results, thereby enhancing productivity. In addition to these benefits, the use of robots to automate manual tasks provides flexibility that typically allows multiple methods and higher throughputs to be realized.

Simulated distillation (SIMDIS) is a gas chromatographic (GC) test method used in the petroleum refining industry. It is used in place of, or in addition to, physical distillation as a way of determining hydrocarbon boiling-point distributions within a sample. The method used depends on the type of sample being analysed. For example, samples with a final boiling-point up to 260°C (500°F) are analysed by ASTM D 3710, while products with a final boiling point up to 538°C (1000°F) are analysed according to ASTM D 2887. Other SIMDIS procedures are available to handle a variety of sample types, particularly for crude oil and higher boiling petroleum materials.

The sample preparation requirements for each SIMDIS method vary. Some require no sample preparation (i.e. neat injection is possible), while others may need heating, dilution(s), addition of internal standard, and more. These latter methods would lend themselves well to automation. A number of articles have described the application of cylindrical laboratory robotics and their advantages to SIMDIS sample prep [2, 3].

### The HP ORCA system

Hewlett-Packard's Optimized Robot for Chemical Analysis (ORCA) system was used to automate a SIMDIS procedure based on ASTM method D 2887 [4]. The HP ORCA system consists of hardware and software that can efficiently automate manual processes. The system provides a number of advantages over cylindrical robots:

(1) The robot is an articulated arm that travels along a linear rail. This configuration results in a virtually rectangular work volume that adapts easily to existing laboratory environments.
(2) The system is expandable. For example, if sample loads increase, resulting in a need to increase the throughput of the robotic system, a longer rail (up to 3 m) and/or the extended envelope option can be added. The extended envelope allows the robot to work optimally on either side of the rail. By adding the above components, the maximum work envelope of 2·98 $m^3$ can be realized.
(3) Standard peripherals can be easily incorporated into the system. The operating software supports industry-standard communication protocols (RS-232 and IEEE-488) that allow interfacing to a wide variety of off-the-shelf devices, from analytical balances to sophisticated analysers. In addition, the flexibility and precision of the robot allow it to physically interact with inexpensive, widely available peripherals, such as standard vial or test-tube racks. In short, costly, specially designed and built peripherals are unnecessary in many cases.
(4) The Microsoft Windows-based software, called Methods Development Software (MDS), is used to control equipment on the bench and communicate automatically with other MS-DOS and Windows applications co-resident on a *single* personal computer.

### Why automate D 2887?

ASTM method D 2887 has traditionally been performed using non-polar packed GC columns. The recent trend has been toward the use of 0·53 mm, thick-film capillary columns, for example, a 10 m × 0·53 mm, 2·65 µm HP-1. In each of these cases, an injection of the neat sample is possible. Consequently, no sample preparation is required. However, this study investigated the possibility of using smaller i.d. (0·32 mm), thinner-film capillary columns. This smaller column provides the following advantages:

(1) Analysis times are decreased.
(2) The maximum temperature required to elute $C_{44}$ is lowered.
(3) The lower bleed characteristics permit a better FBP determination.
(4) The upper boiling range can be easily extended for the analysis of other heavy petroleum fractions.

Using such a column, however, requires sample dilution to avoid overloading the stationary phase. Sample preparation steps such as weighing, liquid dispensing, mixing, and crimp capping are now required. All of these

0142-0453/94 $10.00 © 1994 Taylor & Francis Ltd.

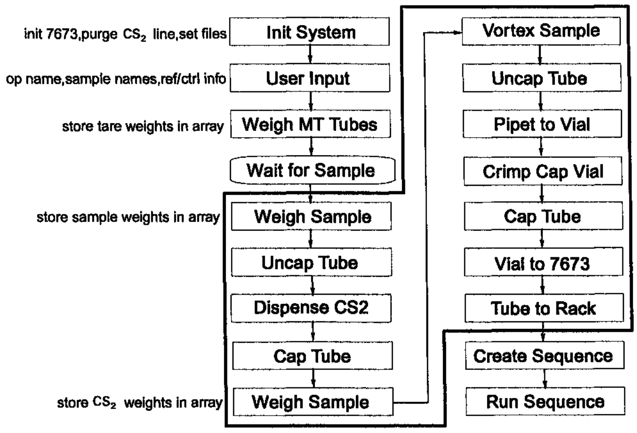

*Figure 1. Flow diagram of the automated SIMDIS sequence. Sample preparation is batched, as shown by the steps in the box.*

tasks are easily handled by the HP ORCA system robot and associated peripherals. Since D 2887 is a relatively straightforward method, it can be used to demonstrate the feasibility of automating other SIMDIS methods.

### The automated SIMDIS process

The automated process is shown in figure 1. ORCA automates all but two tasks in sample preparation and analysis. Manual intervention is currently required to input user and sample information and to qualitatively transfer an aliquot from the original sample container to a capped test-tube that can be manipulated by the robot and peripherals. Sample preparation is currently done in batch mode due to software requirements. Samples ready for analysis are stored in the HP 7673 autosampler tray until the pre-defined number of samples have been prepared by the automated system. At that point, the analysis begins.

The hardware setup is shown schematically in figure 2. MDS manages the entire automated application. It controls all the equipment listed below through industry-standard communication protocols, such as RS-232-C and IEEE-488.

(1) HP G1203A ORCA System with a 2 m rail.
(2) HP G1243A Dispenser System.
(3) HP G1246A Power and I/O Unit.
(4) HP 5890 Series II Gas Chromatograph with Electronic Pressure Control (EPC).
(5) HP 7673 Series II Autosampler.
(6) HP 3488 Switch/Control Unit.
(7) Capping Station with Liquid Dispensing option (Scitec Consultants, Inc., Newark, DE).
(8) Crimp Capping Station (Scitec Consultants, Inc., Newark, DE).
(9) Mettler (Hightstown, NJ) AT261 DeltaRange® analytical balance.
(10) IOtech (Cleveland, OH) Serial 488/4.
(11) Fisher Scientific (Pittsburgh, PA) Vortex Genie 2™.

MDS also acts as the controller of, and the co-ordinator between, two other software packages residing on the PC: the HP 3365 GC ChemStation and AC Analytical Controls Simulated Distillation software [5]. When the

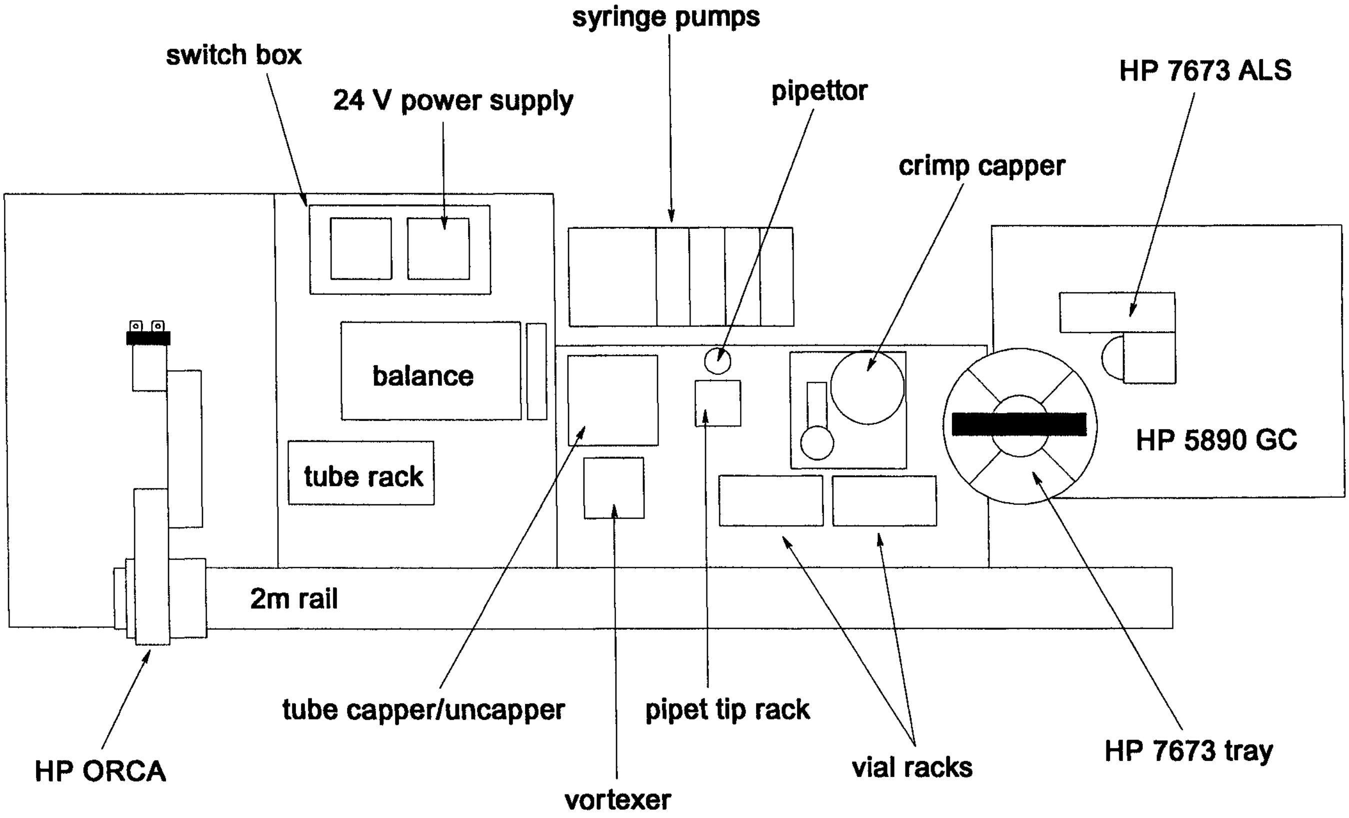

*Figure 2. Schematic representation of the automated SIMDIS application hardware setup.*

*Table 1. The SIMDIS sequence for each batch of samples.*

| Analysis | Name | Description |
|---|---|---|
| 1 | Conditioning | No injection, conditions column |
| 2 | Blank | Column/blank compensation |
| 3 | BP calibration | BP calibration run |
| 4 | Reference | QC sample No. 1 |
| 5 | ASTM RGO | QC sample No. 2 |
| 6 | Sample | Sample analysis |
| 7 | Sample | Sample analysis |
| ⋮ | ⋮ | ⋮ |
| *N* | Reference | QC sample No. 1 |

batch preparation is complete, sample and solvent weights received from the analytical balance, as well as the information entered by the analyst (including operator name, sample name(s), and reference names and weights), are *automatically* downloaded from MDS arrays into an AC SIMDIS sequence created on-the-fly by MDS. This sequence is now available within the HP ChemStation. MDS then loads that sequence through the HP ChemStation and initiates the gas chromatographic analysis. Calculations are handled by the AC software which generates a customized report. The AC software provides graphical reporting of the SIMDIS data by interacting with Microsoft Excel that is also co-resident on the PC.

The AC software was used in this study for a number of reasons. First, it is a Windows 3.1 application that interacts with the HP ChemStation. It can also be controlled by MDS. Second, it is capable of doing multimode SIMDIS calculations, including D 3710 and D 5307 [6] in addition to D 2887. Finally, it allows each SIMDIS sequence to include a number of automated quality control checks. Blanks can be run to determine the baseline and solvent ($CS_2$) compensations and to check for column bleed characteristics; boiling-point calibrations can detect retention time drift and peak skew, as well as verify resolution; and reference samples can check for pre-defined specifications. The SIMDIS sequences created in this work (see table 1) consisted of a column conditioning run (no injection), a blank ($CS_2$) injection, a boiling-point calibrant, two reference samples, the prepared samples, and a repeat of a reference. One of the reference samples (QC sample No. 1) is an AC-supplied reference sample that has been characterized by physical distillation. It is run before and after the samples as a quality control check. An ASTM reference gas oil (RGO) is also used as an internal reference.

### Experimental conditions

Six replicates of two different samples were prepared and analysed manually and robotically. Reference samples were prepared manually for all studies and stored at −22°C until needed. Chromatographic conditions were as follows:

(1) Chromatograph: HP 5890 Series II.

(2) Inlet: EPC cool-on-column (oven track mode).

(3) Injector: HP 7673 Series II with nanoliter adapter and auto 320 injector accessory.

(4) Detector: FID, 350°C.

(5) Column: 7 m × 0·32 mm × 0·5 µm HP-1.

(6) Flow: constant flow He, 15 ml/min.

(7) Temperature program: 35°C initial, 10°C/min, 325°C final (hold 5 min).

(8) Injection volume: 0·5 µl on-column.

(9) Sample dilution: 1% (w/w) calibration mixture in $CS_2$, 5% (w/w) sample in $CS_2$.

### Results

Representative results for each sample are shown in tables 2 and 3. Typical chromatograms are shown in figures 3 and 4. There is excellent agreement between results for the manually and robotically prepared samples. In general, the results from the automated sample preparation show better precision than those obtained from manual preparation. Comparison of the automated results to manual results obtained independent of this work for

*Table 2. Lube base stock: % off versus average boiling-point (°F) ± s (N = 6).*

| % off | Manual prep. B.P. (°F) | Robot prep. B.P. (°F) |
|---|---|---|
| IBP | 580 ± 1 | 589 ± 3 |
| 5 | 651 ± 0 | 653 ± 0 |
| 10 | 676 ± 0 | 676 ± 0 |
| 20 | 703 ± 0 | 705 ± 0 |
| 30 | 723 ± 0 | 723 ± 0 |
| 40 | 739 ± 0 | 739 ± 0 |
| 50 | 755 ± 1 | 756 ± 0 |
| 60 | 770 ± 0 | 770 ± 0 |
| 70 | 788 ± 0 | 788 ± 0 |
| 80 | 808 ± 0 | 808 ± 0 |
| 90 | 839 ± 1 | 838 ± 0 |
| 95 | 868 ± 1 | 867 ± 0 |
| FBP | 979 ± 1 | 979 ± 0 |

*Table 3. Light vacuum gas oil: % off versus average boiling-point (°F) ± s (N = 6).*

| % off | Manual prep. B.P. (°F) | Robot prep. B.P. (°F) |
|---|---|---|
| IBP | 413 ± 2 | 411 ± 0 |
| 5 | 507 ± 1 | 505 ± 0 |
| 10 | 547 ± 0 | 547 ± 0 |
| 20 | 595 ± 0 | 594 ± 0 |
| 30 | 631 ± 0 | 630 ± 0 |
| 40 | 664 ± 1 | 662 ± 0 |
| 50 | 693 ± 0 | 693 ± 0 |
| 60 | 723 ± 0 | 723 ± 0 |
| 70 | 757 ± 0 | 757 ± 0 |
| 80 | 799 ± 1 | 797 ± 0 |
| 90 | 851 ± 0 | 851 ± 0 |
| 95 | 900 ± 1 | 901 ± 0 |
| FBP | 1017 ± 1 | 1019 ± 0 |

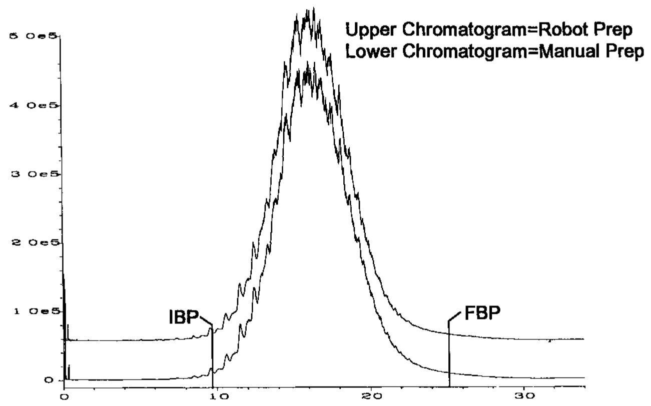

*Figure 3. Comparison of chromatograms (offset to show detail) of lube base stock prepared manually and robotically.*

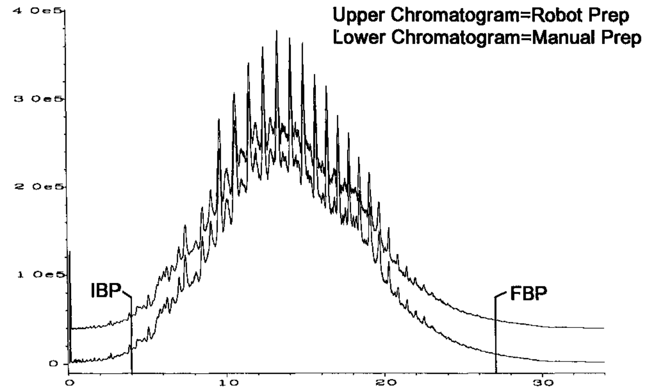

*Figure 4. Comparison of chromatograms (offset to show detail) of light vacuum gas oil prepared manually and robotically.*

*Table 4. Comparison of the results for the AC reference sample (56.40.400) obtained over several days with the actual boiling-points determined by physical distillation (ASTM method D 2892).*

| % off | B.P. (°F) D 2892 | B.P. (°F) Day 1 | B.P. (°F) Day 2 | B.P. (°F) Day 3 | B.P. (°F) Day 4 |
|---|---|---|---|---|---|
| 10 | 406 | 405 | 405 | 405 | 407 |
| 20 | 455 | 453 | 454 | 454 | 455 |
| 30 | 489 | 489 | 489 | 489 | 491 |
| 40 | 520 | 518 | 519 | 519 | 520 |
| 50 | 549 | 547 | 548 | 548 | 549 |
| 60 | 577 | 575 | 576 | 576 | 577 |
| 70 | 606 | 603 | 604 | 604 | 605 |
| 80 | 640 | 635 | 636 | 636 | 636 |
| 90 | 680 | 676 | 676 | 676 | 677 |

the lube base stock also show excellent agreement. Table 4 lists the data obtained for the AC-supplied reference sample (AC No. 56.40.400) over the course of this study. The excellent repeatability is due to the consistent performance of the HP 5890 Series II system and the AC SIMDIS software. Using the chromatographic conditions described above, hydrocarbon boiling-points below $C_{44}$ elute at oven temperatures below 300°C.

### Future directions

Additional work will be done to further automate SIMDIS sample preparation and analysis. Currently, attention will focus on three areas.

### *Remove all manual steps from the current process*

Bar codes can be employed for sample information input. A second tube capping workstation would allow the automated transfer of the sample from the original test-tube to a daughter tube for dilution. To accommodate 'common' sample containers (i.e. other than capped test-tubes), one or more generic automated capping/uncapping workstations could be used. This would allow the robot to pipet and weigh an aliquot of the original sample from a variety of vessel sizes into a tared child container.

### *Expand the scope of the automated application to include other SIMDIS methods, specifically those related to the analysis of viscous or waxy samples*

This can be done by incorporating a heating device into the robotic work space and providing for the addition of internal standard to the samples. The maximum work envelope for the HP ORCA system would allow a single robot to prepare samples and service up to four gas chromatographs, each reserved for a specific method. The Analytical Controls software can perform multiple SIMDIS calculations, including D 3710, extended D 2887, D 5307, and lube oil volatility. Other customized calculations can also be performed. In addition, the AC software coupled to the AC TBP750 is especially suited for the analysis of full range crude oils and heavy resid material.

### *Connect to a LIMS*

This would be beneficial for downloading work lists and GC methods as well as for data archival.

### Conclusions

Results of this work have shown that the HP ORCA system can be used to confidently automate SIMDIS sample preparation and analysis based on ASTM D 2887. Data obtained from the analysis of samples prepared by the robot showed no significant difference as compared to the data obtained from the analysis of manually prepared samples. Extending the applicability of the automated system to other SIMDIS methods should yield similar results.

### Acknowledgements

The authors would like to thank Doug Janson of Analytical Controls for his assistance with the AC SIMDIS software and Wayne Schmidt of Hewlett-Packard for his help with the systems integration.

### References

1. Majors, R. E., *LC/GC*, **9** (1991), 16.
2. Crook, M. J., Deakin, C. J. and Whitmore, B., *Intelligent Instruments and Computers*, March/April (1991), 51.
3. Maynard, J. B. and Michalik, W. A., *Journal of Chromatographic Science*, **26** (1988), 290.
4. D 2887-89, Standard Test Method for Boiling Range Distribution of Petroleum Fractions By Gas Chromatography. *ASTM Annual Book of Standards*, 05.02.
5. Colle, P. and Schaatsbergen, G., *International Laboratory*, **8** (1992),6.
6. D 5307-93, Standard Test Method for Determination of Boiling Range Distribution of Crude Petroleum by Gas Chromatography. *ASTM Annual Book of Standards*, 05.03.

